# Dietary Phytoestrogen Intake and Cognitive Status in Southern Italian Older Adults

**DOI:** 10.3390/biom12060760

**Published:** 2022-05-30

**Authors:** Francesca Giampieri, Justyna Godos, Giuseppe Caruso, Marcin Owczarek, Joanna Jurek, Sabrina Castellano, Raffaele Ferri, Filippo Caraci, Giuseppe Grosso

**Affiliations:** 1 Research Group on Food, Nutritional Biochemistry and Health, European University of the Atlantic, 39011 Santander, Spain; f.giampieri@staff.univpm.it; 2 Department of Biomedical and Biotechnological Sciences, University of Catania, 95123 Catania, Italy; justyna.godos@gmail.com (J.G.); giuseppe.grosso@unict.it (G.G.); 3 Department of Drug and Health Sciences, University of Catania, 95125 Catania, Italy; 4 Research Operative Unit of Neuropharmacology and Translational Neurosciences, Oasi Research Institute-IRCCS, 94018 Troina, Italy; 5 School of Psychology, Ulster University, Coleraine BT52 1SA, UK; owczarek-m@ulster.ac.uk; 6 APC Microbiome Ireland, University College Cork, T12 K8AF Cork, Ireland; jurek-j@ulster.ac.uk; 7 Department of Educational Sciences, University of Catania, 95124 Catania, Italy; sabrina.castellano@unict.it; 8 Sleep Research Centre, Department of Neurology IC, Oasi Research Institute-IRCCS, 94018 Troina, Italy; rferri@oasi.en.it

**Keywords:** phytoestrogens, isoflavones, lignans, genistein, daidzein, cohort, population, cognitive status, cognition, brain

## Abstract

Background: Aging society faces significant health challenges, among which cognitive-related disorders are emerging. Diet quality has been recognized among the major contributors to the rising prevalence of cognitive disorders, with increasing evidence of the putative role of plant-based foods and their bioactive components, including polyphenols. Dietary polyphenols, including phytoestrogens, have been hypothesized to exert beneficial effects toward brain health through various molecular mechanisms. However, the evidence on the association between dietary phytoestrogen intake and cognitive function is limited. The aim of this study was to investigate the association between phytoestrogen intake and cognitive status in a cohort of older adults living in Sicily, Southern Italy. Methods: Dietary information from 883 individuals aged 50 years or older was collected through a validated food frequency questionnaire. Cognitive status was assessed through the Short Portable Mental Status Questionnaire. Results: The highest total isoflavone (including daidzein and genistein) intake was inversely associated with cognitive impairment compared to the lowest (odds ratio (OR) = 0.43, 95% confidence interval (CI): 0.20–0.92). Higher intake of total lignans and, consistently, all individual compounds (with the exception of secoisolariciresinol) were inversely associated with cognitive impairment only in the unadjusted model. Conclusions: A higher intake of phytoestrogens, especially isoflavones, was associated with a better cognitive status in a cohort of older Italian individuals living in Sicily. Taking into account the very low intake of isoflavones in Italian diets, it is noteworthy to further investigate selected populations with habitual consumption of such compounds to test whether these results may be generalized to the Italian population.

## 1. Introduction

Over the last century, there has been a notable increase in life expectancy, especially in the developed countries, contributing to the rise of both the size and the proportion of older people in the global population [1]. An aging society faces significant health challenges and an increase in chronic diseases, including conditions affecting the cognitive function [2]. Non-communicable diseases have become the leading cause of mortality in industrialized nations, as well as the leading cause of disease-related life-years lost worldwide [3]. Among the most important disorders to consider, age-related cognitive decline and cognitive impairment are becoming more common. Additionally, dementias, including both Alzheimer’s disease and vascular dementia, has been shown to be frequent causes of cognitive impairment in older adults across the globe [4]. Interestingly, diet quality, a modifiable risk factor, has been identified among the major contributors to the increasing prevalence of chronic diseases including brain-related disorders [5,6], underlined by systemic low-grade chronic inflammation [7].

Diet plays a key role in both the prevention and improvement of mental status and brain function in the elderly [8,9]. Greater adherence to plant-based dietary patterns and high consumption of foods of plant origin, such as vegetables, fruit, legumes, and whole grains are useful in order to preserve and strengthen cognitive health [10]. The cognitive-related effects of these foods may be attributed to their high content in polyphenols, natural compounds with a chemical structure characterized by hydroxyl groups within aromatic rings [11,12,13]. This vast family of compounds comprises phytoestrogens, such as isoflavones and lignans, which are naturally occurring non-steroidal polyphenols with chemical, structural, and functional similarities to 17β-estradiol [14]. The interaction with estrogen receptors has been shown to regulate gene expression and to modify activities at many levels in the body, reducing the risk of cardiovascular disease, cancer, and osteoporosis [15]. In fact, dietary intake of phytoestrogens has been previously related to several chronic diseases [16,17] and mortality [18]. Phytoestrogens may have a beneficial impact on cognitive performance in older adults, which is linked not only to an increase in production of neurotrophins, but also to their anti-inflammatory and antioxidant activity, hippocampus synaptic regulation, and enhanced nitric oxide bioavailability, which is important for cognitive function maintenance [19,20,21,22,23]. A relatively low number of studies have been conducted to investigate the potential role of phytoestrogen intake on cognitive outcomes in humans, reporting conflicting, yet promising results [24,25,26,27]. Nonetheless, no data have been reported for the Italian population so far. Thus, the aim of this study was to investigate the association between phytoestrogen intake and cognitive status in a cohort of older adults living in Sicily, Southern Italy.

## 2. Materials and Methods

### 2.1. Study Population

The MEAL study is an observational study focusing on the link between nutritional and lifestyle habits typical to the Mediterranean area and non-communicable diseases. The study protocol has been described elsewhere [28]. Briefly, the study enrollment took place between 2014 and 2015 in the main districts of the city of Catania in southern Italy. A sample of 2044 men and women aged 18 or more years old was randomly selected and included in the baseline data. Data collection was carried out using the registered records of local general practitioners stratified by sex and 10-year age groups. In order to provide a specific relative precision of 5% (Type I error, 0.05; Type II error, 0.10), considering an anticipated 70% participation rate, the theoretical sample size was estimated to 1500 individuals.

Out of 2405 individuals invited to participate in the study, 361 refused, leaving 2044 participants (response rate of 85%) as the final sample. Taking into consideration that the investigated outcome is more prevalent in the older population, the analysis for the present study was limited to individuals 50 years of age or older (*n* = 916). All participants were familiarized about goals of the MEAL study and informed written consent was obtained. The study protocol has been refereed and approved by the ethical committee concerned and all the study procedures were conducted in accordance with the Declaration of Helsinki (1989) of the World Medical Association.

### 2.2. Data Collection

A face-to-face assisted personal interview was conducted during which all participants were provided with a paper copy of the questionnaire in order to visualize the response options. However, the final answers were registered directly by the interviewer using tablet computers. Information regarding sociodemographic and lifestyle factors was collected [29]. The sociodemographic data included age at recruitment, sex, and educational status (low (primary/secondary school), medium (high school), and high (university)). Lifestyle variables included physical activity (low, moderate, high; determined by using the International Physical Activity Questionnaire (IPAQ) [30]), smoking status (non-smoker, ex-smoker, and current smoker), and alcohol consumption (none, moderate drinker (0.1–12 g/d), and regular drinker (>12 g/d)).

### 2.3. Dietary Assessment

Aiming to determine the dietary intake, two food frequency questionnaires (FFQ, a long and a short version), previously proved for validity and reliability for the Sicilian population, were adapted [31,32]. Employing the determination of the food intake, the energy content as well as the macro- and micro-nutrient intake was calculated through a comparison with food composition tables of the Italian Research Center for Foods and Nutrition [33]. Intake of seasonal foods refers to consumption during the period in which the food was available and then adjusted by its proportional intake over one year.

Diet quality was evaluated by means of adherence to the Mediterranean diet [34]. Briefly, a literature-based scoring system, which is based on the frequency of intake of different food groups was used. In particular, food groups characteristic to the Mediterranean dietary pattern (such as fruit, vegetables, cereals, legumes, fish, and olive oil) were given positive points, while food groups not representing it (such as meat and dairy products) were given negative points; moderate alcohol intake was deemed as optimal for higher adherence. The final score indicative of adherence to the Mediterranean diet was based on nine food categories with a score ranging from 0 points (lowest adherence) to 18 points (highest adherence), and individuals were clustered in tertiles categorized as low, medium, and high adherence to the Mediterranean diet [35]. FFQs with unreliable intakes (<1000 or >6000 kcal/d) were excluded from the analyses (*n* = 33), leaving a total of 883 individuals included in the analysis.

### 2.4. Estimation of Dietary Phytoestrogen Intake

The process of the estimation of dietary phytoestrogen intake has been previously described in detail [36]. Summarily, data on the phytoestrogen content in plant-based foods and beverages were retrieved from the Phenol-Explorer database [37]. First, the food and beverage consumption was calculated (in g or mL) by following the standard portion sizes used in the study, and then converted to 24 h intake. Afterward, the Phenol-Explorer database was searched to retrieve mean content values for all phytoestrogens contained in the applicable food items included in the FFQ. Phytoestrogen intake from each food was determined by multiplying the content of total, main subclasses, and selected individual phytoestrogens by the daily consumption of each food adjusted for total energy intake (kcal/d) using the residual method [38].

### 2.5. Cognitive Status Evaluation

Cognitive status assessment in the MEAL study was performed using the Short Portable Mental Status Questionnaire (SPMSQ) [39], which was designed to measure cognitive impairment in both the general and hospital population [40], and was previously applied in the Italian population [41]. SPMSQ, a 10-item tool, was administered by the clinician in the office or in a hospital. The pre-defined domains for assessment of the screening tool were the following: (i) intact, less than 3 errors; (ii) mild, 3 to 4 errors; (iii) moderate, 5 to 7 errors, and (iv) severe, 8 or more errors. For this study, we considered 3 or more errors as a cut-off point for impaired cognitive status.

### 2.6. Statistical Analysis

Categorical variables are presented as frequencies of occurrence and percentages; differences between groups were tested with Chi-squared test. Continuous variables are presented as means and standard deviations (SDs) or median and standard errors (SEs) whether they were distributed normally or skewed, respectively; differences between groups were tested with Student’s t-test or Mann–Whitney U-test for variables distributed normally or skewed, respectively. The relation between exposure variables and cognitive status was tested through uni- and multivariate logistic regression analysis adjusted for baseline characteristics (age, sex, BMI, smoking status, alcohol consumption, physical activity level, educational level, and health status). An additional model was performed to further adjust for adherence to the Mediterranean diet, as a proxy variable for diet quality. Subgroup analyses by age groups (70 y as cut-off) were performed to test whether age may act as a confounding factor; given the reduction of the sample, only variables reporting a significant different distribution across tertiles of phytoestrogen intake were included in the multivariate model. All reported *p*-values were based on two-sided tests and compared to a significance level of 5%. SPSS ver. 25 (SPSS Inc., Chicago, IL, USA) software was used for all the statistical calculations.

## 3. Results

**Figure 1:**
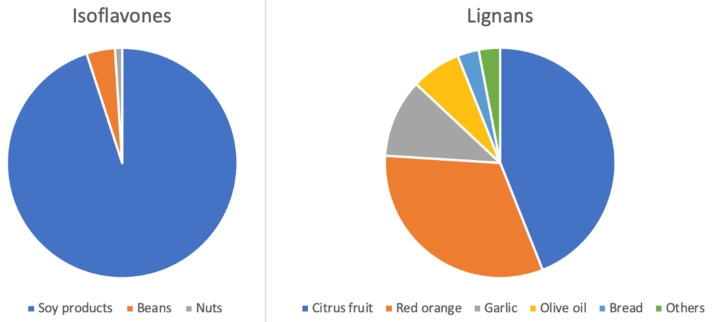
Major food sources of phytoestrogens in the study sample (*n* = 883).

The mean intake of total phytoestrogens in the sample was 5.47 mg/d (median 2.37 mg/d), with roughly half being represented by isoflavones (2.60 mg/d) and half by lignans (2.86 mg/d). The major food sources of isoflavones and lignans are shown in Figure 1. While the main food source of isoflavones were soy products (95%), food sources of lignans were more varied, and mostly represented by citrus fruits and red oranges (Figure 1).

The background characteristics distributed according to phytoestrogen tertiles of intake are presented in Table 1. Individuals consuming less phytoestrogens (lowest tertile of consumption) were significantly older than those consuming more. There was a higher prevalence of females, who were non-smokers with moderate alcohol consumption, among those in the highest tertile of phytoestrogen intake. Adherence to the Mediterranean diet was distributed differently across groups of phytoestrogen intake, being an increasing proportion of high adherent individuals with higher compared to lower intake of phytoestrogens.

Out of the 883 participants included in the study, 9.1% (*n* = 83) were identified as cognitively impaired. These individuals were significantly older (64.3 y vs. 70.3 y cognitively normal vs. impaired, respectively) and less active (24.2% vs. 43.4% physically inactive among cognitively normal vs. impaired, respectively). The total median intake of specific phytoestrogens and the differences between cognitive healthy and impaired participants are presented in Table 2. Participants with cognitive impairment resulted in having a significantly lower intake of matairesinol with no other substantial differences evident in this analysis.

The association between specific phytoestrogen intake and cognitive status is reported in Table 3. The highest isoflavone intake compared to the lowest was inversely associated with cognitive impairment in the multivariate analysis (odds ratio (OR) = 0.43, 95% confidence interval (CI): 0.20–0.92) but it resulted in being sensibly weakened when further adjusted for adherence to the Mediterranean diet (OR = 0.46, 95% CI: 0.21–1.00). When exploring the individual isoflavone compounds, higher intake of both daidzein (OR = 0.44, 95% CI: 0.21–0.92) and genistein (OR = 0.38, 95% CI: 0.18–0.78) were significantly inversely associated with cognitive impairment compared to lower intake in all adjustment models. Higher intake of total lignans and, consistently, all individual compounds (with exception for secoisolariciresinol) were inversely associated with cognitive impairment in the less adjusted model, but no significant results were obtained after adjustment for potential confounding factors (Table 3).

Since age was related to both the exposure (phytoestrogen intake) and the outcome (cognitive status), and may represent a potential confounding factor, we further performed a sensitivity analysis by age groups, exploring the potential role of major phytoestrogen group consumption in the elderly population (≥70 years old). After adjustment for other covariates, older individuals reporting a higher intake of both isoflavones and lignans were less likely to be cognitively impaired compared to lower consumers (OR = 0.10, 95% CI: 0.01–0.88 and OR = 0.16, 95% CI: 0.03–0.87, respectively; Table 4). No significant results for the younger group were found.

## 4. Discussion

The results of this study showed a significant association between total isoflavone intake and, more specifically, daidzein and genistein, and cognitive status in Sicilian adults. When stratifying by age, the results were stronger among older individuals, including an association between higher lignan intake and lower odds of cognitive impairment. These findings add up to previous observational studies exploring the effect of phytoestrogen consumption on cognitive health, although current evidence is rather conflicting. A previous study conducted on a cohort of 403 men and 373 women aged 60–81 years from the National Institute for Longevity Sciences and their Longitudinal Study of Aging conducted in Japan, reported similar findings, with isoflavone consumption associated with decreased risk of cognitive decline, although only in women [24]. The authors hypothesized that the estrogen levels’ receptors in the central nervous system may play some role on memory and cognitive performance, and that post-menopausal women may benefit from isoflavone consumption better than men. In contrast, the Singapore Chinese Health Study reported null association between dietary isoflavone intake and risk of cognitive impairment among 16,948 participants followed for over 15 years [25]. However, these findings have to be considered with caution: this cohort has been reported to have a substantial high intake of isoflavone (such as about 6 mg/d vs. 32 mg/d median intake in the lowest vs. the highest category of consumption, respectively); compared to those reported in the present cohort (roughly 0 mg/d vs. 9 mg/d in the lowest vs. the highest category of consumption, respectively), these intakes may not capture the real role of isoflavone in the diet compared to lack of intake, as even those with minimum consumption had almost as much isoflavone intake as those in the highest category of exposure in a Western cohort. Results from a community-based survey conducted among 394 post-menopausal Dutch women (mean age: 66.3 years) from a PROSPECT study suggested that a higher dietary intake of lignans is associated with a better cognitive performance, only among women with a long post-menopausal time span [42], while dietary intake of isoflavones is not related to cognitive function [42]. Similarly, a cross-sectional study conducted on 301 Dutch women aged 60–75 years, found no association between isoflavone intake and cognitive function, yet high lignan intake was associated with a better performance in processing capacity and speed, and in executive function [26]. The National Health and Nutrition Examination Survey that included 354 individuals aged 65–85 years old showed significant gender differences in the relationship between genistein and speed of processing (SOP), and higher levels of genistein were associated with better SOP in women but worse in men [27]. Similarly, results from the National Health and Nutrition Examination Survey from a sample consisting of 200 older women (mean age: 74.4 years) suggested that it is crucial to consider and clearly define the levels of daily intake of phytoestrogens to properly determine the association between their intake and cognitive function [43]. Finally, the Study of Women’s Health Across the Nation (SWAN), a multiethnic, community-based, longitudinal study of women aged 42 to 52 years at entry, found no associations between dietary genistein intake and cognitive performance [44], suggesting that future studies should take into consideration not only the dietary intake, but also the bioavailability and actual exposure to the phytoestrogen metabolites [45]. A more recent report from the SWAN cohort on women undergoing menopausal transition confirmed that the association between dietary phytoestrogens and cognition is multifactorial and may depend on the evaluated compound and cognitive domain as well as ethnic groups [46]. Indeed, a recent cross-sectional analysis of 152 individuals (mean age: 69.2 years) divided into two groups based on the equal production status, suggested that equal production status may mediate the relation between dietary isoflavone intake and cognitive function [47]. In summary, studies conducted so far have not properly addressed the issue and reported no consistent evidence. No specific aspect has been identified to explain such contrasting findings, while in the reviewed studies, it has been hypothesized that unmeasured variables, such as genetics and gut microbiota, may be responsible for the interindividual variability of the findings, and need to be further investigated.

From a mechanistic point of view, neurodegenerative disorders share common mechanisms with other chronic diseases, underlined by an increase in oxidative stress and inflammation acting as activators of downstream pathways affecting homeostasis [48,49]. The evidence from experimental studies suggested that genistein exerts both neuroprotective and pro-cognitive activity [50]. Genistein has been shown to increase the expression and secretion of BDNF and NGF neurotrophic factors along with the glial-derived neurotrophic in cultured astrocytes [51]. In animal models, dietary genistein has been demonstrated to be able to decrease the brain oxidative stress induced by stroke in rats, by decreasing the activity of NADPH oxidase enzyme, to reduce superoxide levels, [52] and to decrease ROS and MDA formation, by enhancing antioxidant enzyme activities (SOD and glutathione peroxidase), reversing the mitochondria dysfunction, and suppressing phosphorylation and activation of the NF-κB p65 subunit, and the phosphorylation and degradation of the inhibitor protein of κBα (IκBα) [53]. Similarly, daidzein has neuroprotective effects through ligand-binding-independent per-oxisome proliferator-activated receptor gamma (PPARγ) activation and inhibition of NF-κB signaling [54]. The ability of daidzein, as well as other isoflavones such as genistein and equol to protect and modulate PPARγ, has also been observed in both the periphery (macrophages) and astrocytes [55]. Part of the neuroprotection exerted by daidzein and its metabolites could be due to their antioxidant properties being stronger than those of other isoflavones such as formononetin and biochanin A [54]. Regarding lignans, several in vitro and in vivo studies explored whether lignans may play a role in neuroprotection and cognitive function. Similarly, to other polyphenol classes, lignans have been demonstrated to exert anti-oxidative and anti-inflammatory properties in neurons as well as inhibit LPS-activated microglia [56]. This class of polyphenols has been shown to improve memory impairment and facilitate hippocampal LTP induction via blocking acetylcholinesterase (AChE), an acetylcholine-degrading enzyme, and improving calcium influx into neuronal cells, as well as [57] reducing oxidative stress, inflammation, and blood–brain barrier permeability [58]. In the present study, we did not find any significant association between lignan intake and cognitive status. We may hypothesize that dietary sources of lignans in this cohort did not provide the adequate amount required to exert any potential risk modification, or that some specific molecules otherwise not yet identified were to be the ones actually providing protective effects, if any. However, currently, there are no human trials using supplements or dietary modulation to increase total daily lignan intake; thus, the observed results retrieved from pre-clinical studies may not apply to humans.

Based on the data presented in this study, most dietary isoflavones consumed by the participants were derived from soy-based products. Soy consumption in Italy can be considered relatively low when compared to Asian countries, and also the UK or other central and north European countries, although comparable or higher than the remaining southern European countries [59]. As several other non-Asian countries, soy is often provided via soy-based meat and dairy alternatives [60]. Although clinical studies on the effects of soy consumption and cognitive health are lacking, inclusion of soybeans in the diet as a meat substitute has been shown to improve systemic inflammatory markers in diabetic patients [61]. In fact, use of plant-based meat substitutes has been suggested not to lead to substantial changes of overall diet quality, and nutrient adequacy and security [62]. Implementing the consumption of soy isoflavones in countries with no tradition of use for this type of food is the object of recent research also regarding environmental aspects related to diet [63]. Although plant-based meat alternatives have always been considered by vegetarian individuals, nowadays, the rising concerns toward greenhouse gas emissions, land occupation, and water consumption provide the rationale for a larger potential share of “health and environmental conscious” new consumers [64]. Inclusion in the diet of soy-based meat and dairy alternatives is not without criticism, as these products mostly fall into the category of ultra-processed foods, which are under the radar for their relation with health [65]. Specifically, these types of foods are hypothesized to exert detrimental effects on health due to nutritional [66] and, most importantly, non-nutritional factors, such as chemical additives [67]. Although several meta-analyses have been published reporting an increased risk of several non-communicable diseases [68,69], research is still ongoing, and the debate whether the benefits would justify the risks is far to be solved. Noteworthy, soy-based meat and dairy alternatives may be implemented in non-Asian countries without requiring modification of meal patterns or food habits by definition, thus resulting in a potential high compliance and ease in reaching population-level targets.

The findings of this study should be considered in light of some limitations. Data on phytoestrogens are derived from FFQs, which are widely used in nutritional epidemiology but also share a number of limits, such as recall bias, and overestimation of intakes for FFQ with many food items or underestimation of intakes due to patient social desirability and social approval bias. Moreover, the FFQs were not specifically designed to estimate the phytoestrogen content, despite having been validated for most food groups representing their major dietary sources. Another limitation relies on the assessment of cognitive status, as the SPMSQ is best suited to be used as a screening tool; however, although a battery of neuropsychological tests could be more suitable to evaluate cognitive performance, it is difficult to implement in practice, and screening tools are widely used to assess the cognitive status rather than performance. Finally, although we adjusted for several potential confounding factors, unmeasured or unaccounted variables may still play a role in the association between dietary exposure and the outcomes assessed. The study is observational in its nature, and we cannot define a cause–effect relationship, only associations.

## 5. Conclusions

In conclusion, moderate intake of phytoestrogens and, specifically, a higher intake of isoflavones were associated with a better cognitive status in a cohort of older Italian individuals living in Sicily. Taking into account the rather low intake of isoflavones in Italian diets, it is noteworthy to further investigate selected populations with habitual consumption of such compounds to test whether these results may be generalized to the Italian population.

## Figures and Tables

**Table 1:** Background characteristics by tertiles of dietary isoflavone and lignan intake in the study sample (*n* = 883).

	Phytoestrogen Intake			
	T1, *n* = 270 (Median = 1.0 mg/d)	T2, *n* = 327 (Median = 2.3 mg/d)	T3, *n* = 286 (Median = 6.2 mg/d)	*p*-Value
**Age (years), mean (SD)**	66.2 (11.1)	64.9 (9.4)	63.8 (7.9)	0.011
**Sex, n (%)**				0.008
Male	110 (40.7)	163 (49.8)	109 (38.1)	
Female	160 (59.3)	164 (50.2)	177 (61.9)	
**Weight status, n (%)**				0.067
Normal	111 (42.4)	103 (33.1)	86 (33.3)	
Overweight	104 (39.7)	130 (41.8)	105 (40.7)	
Obese	47 (17.9)	78 (25.1)	67 (26.0)	
**Educational level, n (%)**				0.132
Low	125 (46.3)	174 (53.2)	152 (53.1)	
Medium	104 (38.5)	95 (29.1)	86 (30.1)	
High	41 (15.2)	58 (17.7)	48 (16.8)	
**Smoking status, n (%)**				0.042
Non-smoker	156 (57.8)	175 (53.5)	166 (58.0)	
Ex-smoker	55 (20.4)	62 (19.0)	70 (24.5)	
Current smoker	59 (21.9)	90 (27.5)	50 (17.5)	
**Physical activity, n (%)**				0.198
Low	63 (27.0)	67 (24.0)	66 (27.8)	
Medium	120 (51.5)	130 (46.6)	120 (50.6)	
High	50 (21.5)	82 (29.4)	51 (21.5)	
**Alcohol intake, n (%)**				<0.001
No	57 (21.1)	77 (23.5)	56 (19.6)	
Moderate	162 (60.0)	207 (63.3)	146 (51.0)	
Regular	51 (18.9)	43 (13.1)	84 (29.4)	
**Mediterranean diet adherence, n (%)**				<0.001
Low	255 (94.4)	264 (80.7)	230 (80.4)	
High	15 (5.6)	63 (19.3)	56 (19.6)	
**Health status, n (%)**				
Hypertension	205 (75.9)	248 (75.8)	207 (72.4)	0.533
Cardiovascular disease	127 (47.0)	138 (42.4)	141 (49.3)	0.195
Cancer	34 (12.6)	23 (7.0)	17 (5.9)	0.01
**Total energy intake (kcal/d), mean (SD)**	1855.1 (543.5)	2042.9 (558.4)	2234.8 (780.3)	<0.001

**Table 2:** Average total phytoestrogen and individual compound intake in the sample, total and by cognitive status.

	Total Population	Cognitively-Healthy	Cognitively- Impaired	*p*-Value
		*Mean (SD)*		
**Phytoestrogens (mg/day)**	5.47 (10.72)	5.51 (10.63)	5.05 (11.68)	0.709
**Isoflavones (mg/day)**	2.61 (10.18)	2.60 (10.14)	2.67 (10.63)	0.953
**Daidzein (mg/day)**	0.09 (0.22)	0.09 (0.22)	0.09 (0.25)	0.816
**Genistein (mg/day)**	0.09 (0.26)	0.09 (0.26)	0.09 (0.29)	0.853
**Lignans (mg/day)**	2.86 (2.56)	2.91 (2.58)	2.38 (2.28)	0.071
**Lariciresinol (mg/day)**	1.54 (1.55)	1.57 (1.56)	1.25 (1.38)	0.075
**Matairesinol (mg/day)**	0.03 (0.03)	0.03 (0.03)	0.03 (0.03)	0.041
**Pinoresinol (mg/day)**	0.99 (0.80)	1.01 (0.81)	0.85 (0.72)	0.099
**Secoisolariciresinol (mg/day)**	0.12 (0.09)	0.12 (0.09)	0.10 (0.09)	0.068

**Table 3:** Association between total and individual isoflavone and lignan intake, and impaired cognitive status.

	Phytoestrogen Intake		
	T1	T2	T3
**Phytoestrogens (mg/d), mean (SD)**	1.00 (0.36)	2.56 (0.69)	13.01 (16.42)
Model 1, OR (95% CI) ^a^	1	0.42 (0.23–0.75)	0.70 (0.40–1.22)
Model 2, OR (95% CI) ^b^	1	0.44 (0.22–0.86)	1.16 (0.61–2.19)
Model 3, OR (95% CI) ^c^	1	0.48 (0.24–0.94)	1.25 (0.65–2.41)
**Isoflavones (mg/d), mean (SD)**	0.01 (0.01)	0.05 (0.02)	9.05 (17.49)
Model 1, OR (95% CI) ^a^	1	0.67 (0.39–1.13)	0.57 (0.30–1.06)
Model 2, OR (95% CI) ^b^	1	0.61 (0.34–1.11)	0.43 (0.20–0.92)
Model 3, OR (95% CI) ^c^	1	0.66 (0.36–1.22)	0.46 (0.21–1.00)
**Daidzein (mg/d), mean (SD)**	0.01 (0.00)	0.03 (0.01)	0.22 (0.34)
Model 1, OR (95% CI) ^a^	1	0.77 (0.45–1.32)	0.52 (0.28–0.95)
Model 2, OR (95% CI) ^b^	1	0.76 (0.42–1.40)	0.41 (0.20–0.84)
Model 3, OR (95% CI) ^c^	1	0.84 (0.45–1.57)	0.44 (0.21–0.92)
**Genistein (mg/d), mean (SD)**	0.00 (0.00)	0.02 (0.01)	0.23 (0.41)
Model 1, OR (95% CI) ^a^	1	0.60 (0.35–1.03)	0.49 (0.27–0.88)
Model 2, OR (95% CI) ^b^	1	0.50 (0.27–0.94)	0.36 (0.18–0.73)
Model 3, OR (95% CI) ^c^	1	0.53 (0.28–1.01)	0.38 (0.18–0.78)
**Lignans (mg/d), mean (SD)**	0.85 (0.34)	1.92 (0.41)	5.28 (2.75)
Model 1, OR (95% CI) ^a^	1	0.64 (0.37–1.11)	0.48 (0.26–0.88)
Model 2, OR (95% CI) ^b^	1	0.71 (0.38–1.32)	0.74 (0.37–1.47)
Model 3, OR (95% CI) ^c^	1	0.77 (0.40–1.46)	0.84 (0.41–1.71)
**Lariciresinol (mg/d), mean (SD)**	0.35 (0.19)	0.98 (0.23)	2.99 (1.69)
Model 1, OR (95% CI) ^a^	1	0.51 (0.30–0.90)	0.44 (0.24–0.80)
Model 2, OR (95% CI) ^b^	1	0.54 (0.29–1.02)	0.66 (0.34–1.29)
Model 3, OR (95% CI) ^c^	1	0.58 (0.30–1.11)	0.73 (0.36–1.47)
**Matairesinol (mg/d), mean (SD)**	0.01 (0.00)	0.02 (0.01)	0.06 (0.04)
Model 1, OR (95% CI) ^a^	1	0.80 (0.47–1.37)	0.52 (0.28–0.98)
Model 2, OR (95% CI) ^b^	1	0.84 (0.44–1.57)	0.80 (0.39–1.62)
Model 3, OR (95% CI) ^c^	1	0.90 (0.48–1.70)	0.92 (0.44–1.91)
**Pinoresinol (mg/d), mean (SD)**	0.34 (0.12)	0.70 (0.14)	1.76 (0.84)
Model 1, OR (95% CI) ^a^	1	0.66 (0.38–1.14)	0.48 (0.26–0.89)
Model 2, OR (95% CI) ^b^	1	0.73 (0.39–1.36)	0.75 (0.38–1.47)
Model 3, OR (95% CI) ^c^	1	0.80 (0.42–1.51)	0.85 (0.42–1.71)
**Secoisolariciresinol (mg/d), mean (SD)**	0.04 (0.02)	0.09 (0.02)	0.21 (0.10)
Model 1, OR (95% CI) ^a^	1	0.68 (0.39–1.18)	0.62 (0.34–1.12)
Model 2, OR (95% CI) ^b^	1	0.69 (0.36–1.32)	1.03 (0.52–2.04)
Model 3, OR (95% CI) ^c^	1	0.77 (0.40–1.49)	1.18 (0.58–2.38)

^a^ Model 1 adjusted for energy intake (kcal/day, continuous); ^b^ Model 2 = Model 1 additionally adjusted for age (continuous), sex (categories), BMI (categories), smoking status (smokers, ex-smokers, non-smokers), alcohol consumption (0 g/day, <12 g/day, ≥12 g/day), physical activity level (low, medium, high), educational level (low, medium, high), and health status; ^c^ Model 3 = Model 2 + adherence to the Mediterranean diet (categories). OR (odds ratio); CI (confidence interval).

**Table 4:** Association between total isoflavone and lignan intake, and impaired cognitive status by age groups.

	Phytoestrogen Intake		
	T1	T2	T3
**Phytoestrogens (mg/d), mean (SD)**	1.03 (0.3)	2.55 (0.71)	13.27 (17.04)
<70 y, OR (95% CI) ^a^	1	1.01 (0.39–2.61)	1.99 (0.87–4.53)
**Phytoestrogens (mg/d), mean (SD)**	0.96 (0.37)	2.59 (0.65)	12.03 (14.00)
≥70 y, OR (95% CI) ^a^	1	0.41 (0.15–1.11)	0.42 (0.09–1.89)
**Isoflavones (mg/d), mean (SD)**	0.01 (0.01)	0.05 (0.01)	7.58 (14.68)
<70 y, OR (95% CI) ^a^	1	0.61 (0.26–1.39)	0.79 (0.35–1.77)
**Isoflavones (mg/d), mean (SD)**	0.01 (0.01)	0.05 (0.02)	9.54 (18.35)
≥70 y, OR (95% CI) ^a^	1	0.52 (0.19–1.44)	0.10 (0.01–0.88)
**Lignans (mg/d), mean (SD)**	0.85 (0.33)	1.89 (0.41)	5.59 (3.02)
<70 y, OR (95% CI) ^a^	1	1.18 (0.49–2.88)	1.68 (0.71–4.00)
**Lignans (mg/d), mean (SD)**	0.85 (0.34)	1.98 (0.39)	4.28 (1.06)
≥70 y, OR (95% CI) ^a^	1	0.79 (0.31–2.05)	0.16 (0.03–0.87)

^a^ Multivariate model adjusted for energy intake (kcal/day, continuous), sex (categories), smoking status (smokers, ex-smokers, non-smokers), alcohol consumption (0 g/day, <12 g/day, ≥12 g/day), physical activity level (low, medium, high), and adherence to the Mediterranean diet (categories). OR (odds ratio); CI (confidence interval).

## Data Availability

The data that support the findings of this study are available upon reasonable request.
